# Early Growth and Physiological Acclimation to Shade and Water Restriction of Seven Sclerophyllous Species of the Mediterranean Forests of Central Chile

**DOI:** 10.3390/plants13172410

**Published:** 2024-08-29

**Authors:** Marco A. Yáñez, Sergio E. Espinoza, Carlos R. Magni, Eduardo Martínez-Herrera

**Affiliations:** 1 College of Forestry, Agriculture, and Natural Resources, University of Arkansas at Monticello, 110 University Ct., Monticello, AR 71656, USA; yanez@uamont.edu; 2 Departamento de Ciencias Forestales, Facultad de Ciencias Agrarias y Forestales, Universidad Católica del Maule, Avenida San Miguel 3605, Talca 3460000, Chile; espinoza@ucm.cl; 3 Centro Productor de Semillas y Árboles Forestales (CESAF), Facultad de Ciencias Forestales y de la Conservación de la Naturaleza, Universidad de Chile, Avenida Santa Rosa 11365, La Pintana 8820808, Chile; crmagni@uchile.cl

**Keywords:** shade tolerance, drought tolerance, sclerophyllous species, Mediterranean climates, forest restoration

## Abstract

The success of using active restoration in Mediterranean-type climate zones mostly depends on an appropriate matching of plant species and specific management prescriptions upon establishment. In this study, we assessed the early growth and short-term physiological acclimation of seven common species found in the sclerophyllous forests in central Chile to water restriction and shading. We established a nursery experiment that included three treatments (T0: sun-exposed and water-restricted, T1: sun-exposed and fully irrigated, and T2: shaded and fully irrigated) and seven tree species differing in their shade and drought tolerance (*Quillaja saponaria* Molina, *Aristotelia chilensis* (Mol.) Stuntz, *Peumus boldus* Molina, *Lithraea caustica* (Mol.) Hook. and Arn, *Luma apiculata* (DC.) Burret, *Colliguaja odorifera* Molina, and *Escallonia pulverulenta* (Ruiz and Prav.) Pers). We measured the increment in seedling height and different leaf morpho-physiological traits during two months in the dry season. Based on the measured traits, none of the species took advantage of the higher water availability in T1 relative to T0, but most of the species responded to the shade in T2, regardless of their shade or drought tolerance. Height increments due to shade varied from 0% in *P. boldus* to 203% in *L. apiculata*. Overall, all the species responded similarly to the treatments in specific leaf area, chlorophyll content index, photosynthetic rate, stomatal conductance, and intrinsic water use efficiency. This suggests that the species exhibited similar acclimation patterns of these parameters to shade and drought, even regarding the variation in midday xylem water potential found in the water-restricted treatment T0 (from −1.5 MPa in *P. boldus* to −3.1 MPa in *E. pulverulenta*). In this study, shading had a higher positive effect on the seedling performance of sclerophyllous species than watering, which at operational level highlights the need for investing in tree shelters when using these species in restoration programs.

## 1. Introduction

The more intense and prolonged droughts due to climate change are threatening the recruitment of young seedlings in Mediterranean ecosystems [1]. Assisted reforestation (i.e., active restoration) is seen as the main strategy to speed up the recovery of forest functions and the structure of degraded zones in this ecosystem type. However, there is still a lack of knowledge on specific management prescriptions for successful seedling establishment and survival in restoration projects, which emphasizes the importance of screening in controlled experiments (i.e., at the nursery level) before scaling projects at the landscape level. Overall, seedlings are more susceptible to drought than adult trees. Thus, information at the seedling stage might give indications on the capacity of a species to grow and survive under environmental stresses and provide guidelines for specific management prescriptions at establishment.

Central Chile possesses one of the five Mediterranean-type climate zones in the world, corresponding to the transition between the Atacama Desert in the north and the mixed deciduous–evergreen temperate forests in the southern part of the country [2]. This zone is considered a hotspot of biodiversity, characterized by its high richness and endemism of plant species [3] but is also considered one of the most degraded ecosystems in the country by anthropogenic disturbances [4]. Overall, the semi-arid region is extensively dominated by sclerophyllous forest species (i.e., matorral shrublands) [5,6]. In this ecosystem, sclerophyllous plant species are adapted to the cool wet winters and warm dry summers historically found in this zone, with many species presenting leathered and small leaves, shrubby habits, and evergreenness [7]. However, despite these adaptation attributes, in recent years, a large-scale browning of this ecosystem type due to the desiccation of vegetation has been observed [8], which has been associated with the mega-drought reported since 2010 [9]. This phenomenon is reducing the canopy cover and creating new microenvironments at the understory level, which threatens the recruitment and regeneration of sclerophyllous species, mainly because young seedlings are unable to establish and survive under the new conditions of high radiation and water scarcity [10,11,12]. In addition, the future intensification of droughts, combined with higher temperatures, also jeopardizes the success of restoration efforts because severe drought and high levels of irradiance during summer severely constrain seedling establishment [11]. Therefore, understanding the responses to drought and high irradiance in sclerophyllous species at the early stages of development is urgently needed as it may provide knowledge to guide the design of species-specific establishing prescriptions.

Most of the success in active restoration relies on seedling survival during the first years after establishment [13,14], where small seedlings struggle with the transplant shock. This is especially the case of Mediterranean-type clime zones, such as the one in central Chile, where post-planting seedling survival depends mainly on the soil water availability, the susceptibility of different plant species to drought, and the effectiveness of the establishment prescriptions addressed to ameliorate that stress. In this context, irrigation of seedlings during the first growing seasons in this climate type has been found to be crucial for improving survival and growth [11,12,15]. Depending on the duration and intensity of drought, plant mortality is caused due to a dysfunction of the xylem hydraulic conductivity (i.e., hydraulic failure) and through the stomatal closure, which negatively affects the carbon balance (i.e., carbon starvation) [16,17]. In this regard, plant species have different mechanisms to cope with drought (i.e., escaping, avoidance, and tolerance) [18,19], but their response to irrigation is still poorly understood in sclerophyllous species. The few studies addressing the responses of sclerophyllous species of central Chile to drought have been mostly carried out on few species under different experimental conditions [20,21,22,23,24], which complicates the comparison between species and studies. Thus, there is a need to expand this type of research to a higher number of species [25] since the restoration of this type of ecosystem must be addressed toward the development of mixed forests.

Excess irradiation and temperature may also exacerbate water stress [10,13], leading plants to their physiological limits determining growth and survival [26]. Plant species have different adaptations to cope with the light spectrum in their lifetime. Depending on their capacity to develop and grow in environments with low light, they are classified as shade-tolerant or intolerant species [27]. Regardless of the shade tolerance of sclerophyllous species, shading by using any type of tree shelter has become a more common practice in restoration projects in Mediterranean-type climates, improving seedlings’ performance [13]. Operationally, and depending on the shelter type, shading improves the microclimate conditions that lead to lower evaporative demand [28], such as lower air temperatures and higher humidity [13]. The acclimation of plants to shade includes a change in stem slenderness (higher height and lower diameter growth) [29]; a decrease in root growth in shade-intolerant species but not in shade-tolerant species [30]; a decrease in photosynthetic rate, stomatal conductance, and photochemical efficiency [31,32,33]; and an increase in specific leaf area (SLA) and chlorophyll content [27,34]. However, under water deficit, plant short-term responses to shading are species-specific [31,35,36,37] since the acclimation responses to light and water restriction seem to be uncoupled for some species (i.e., no trade-off) [38,39]. 

In general, restoration programs in Mediterranean-type climates are not driven economically because most of the species are not of commercial interest. Moreover, management prescriptions such as irrigation and the use of shelters may be very expensive, both operationally and experimentally, which delays the effort in the ecosystem restoration. Thus, the screening of species in controlled conditions at the nursery level is encouraged. In this study, we established a nursery experiment with seven sclerophyllous forest species commonly found in the Mediterranean zone of central Chile, which differ in their shade tolerance. We hypothesized that the species differed in their response to shade and water restriction and that the combined effect of watering and shade will favor the shade-tolerant more than intolerant–intolerant species. In this context, the objective of this study was to assess the growth and short-term physiological acclimation of the species to water restriction and shading. This type of study may help to improve the prescriptions for seedling establishment in restoration programs in Mediterranean zones.

## 2. Results

**Figure 1:**
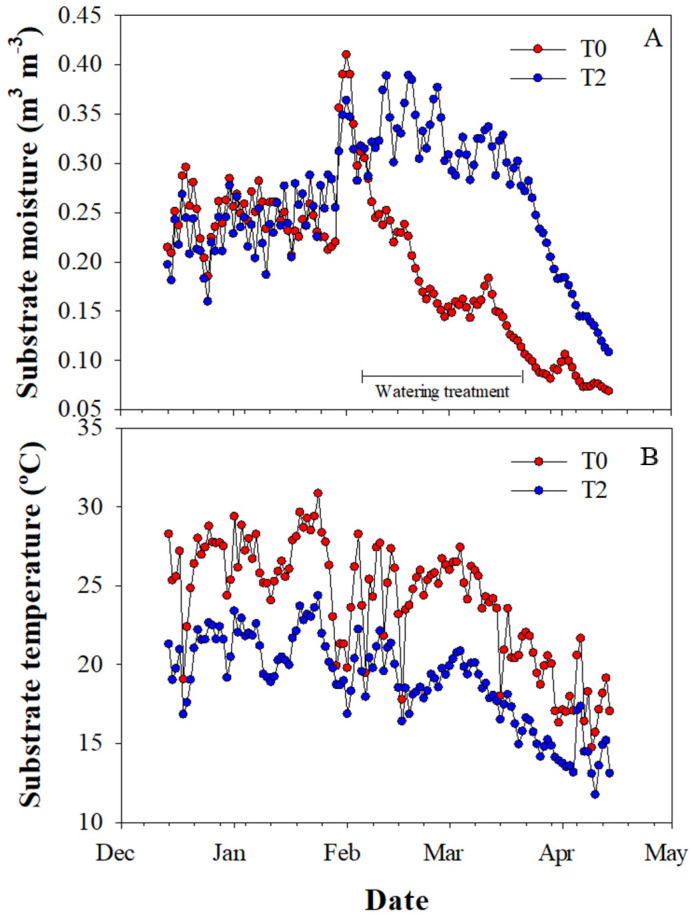
Daily mean moisture (**A**) and temperature (**B**) of the substrate for the full (T2) and restricted (T0) water treatments during the experimental period.

The water restriction was successfully imposed for two months during the experiment, with the substrate moisture content in a range of 0.10–0.26 m^3^ m^−3^ and 0.27–0.39 m^3^ m^−3^ for T0 and T2, respectively (Figure 1A). Additionally, conditions in treatment T2 (shading and irrigation) led to a considerably decrease in the substrate temperature during the experiment (10 °C less, approximately) when compared to T0 (Figure 1B). 

**Figure 2:**
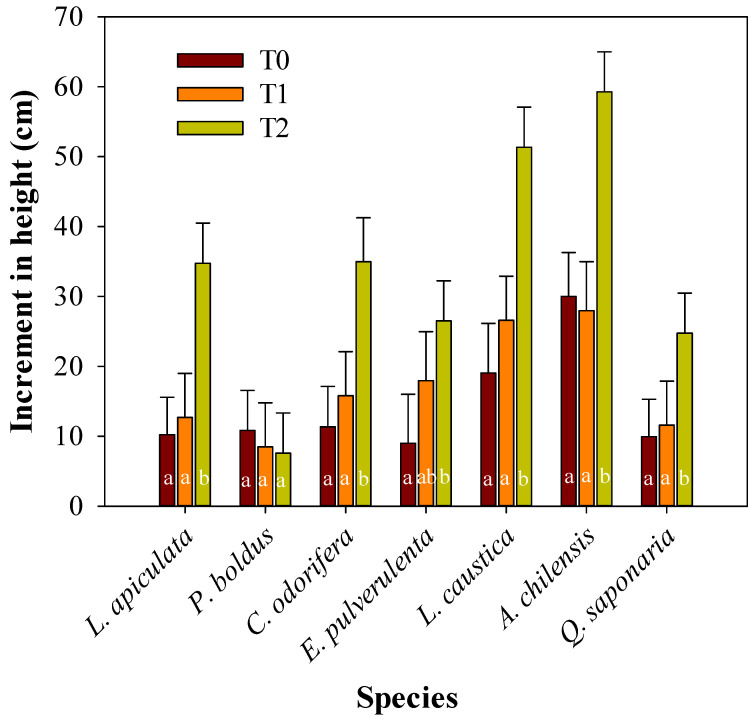
Mean height increment during the experimental period per plant species and treatment (T0, T1, and T2). Different letters denote significant differences among treatments for a specific plant species according to Tukey’s test, with a significance level of 0.05.

Overall, seedling survival by species in the experiment was 100% for *L. apiculata*, and *Q. saponaria*, 94% for *P. boldus* and *C. odorifera*, 83% for *L. caustica* and *A. chilensis*, and 74% for *E. pulverulenta*. At the treatment level, survival was 98% for T2, 97% for T1, and 80% for T0. The analysis of variance showed a significant interaction between treatments and species for the height increments (Table 1), implying a differential response of the species to the treatments (Figure 2). In the experimental conditions, none of the species improved their height growth by the extra amount of water added, as expected (treatment T0 versus T1). Nonetheless, all the species had significant increments in height in T2, except *P. boldus* (Figure 2). The height increment responses of the species in T2 relative to the average of the other treatments were, from high to low, *L. apiculata* (203%), *C. odorifera* (158%), *Q. saponaria* (130%), *L. caustica* (125%), *A. chilensis* (104%), *E*. *pulvurulenta* (97%), and *P. boldus* (0%) (Figure 2).

**Figure 3:**
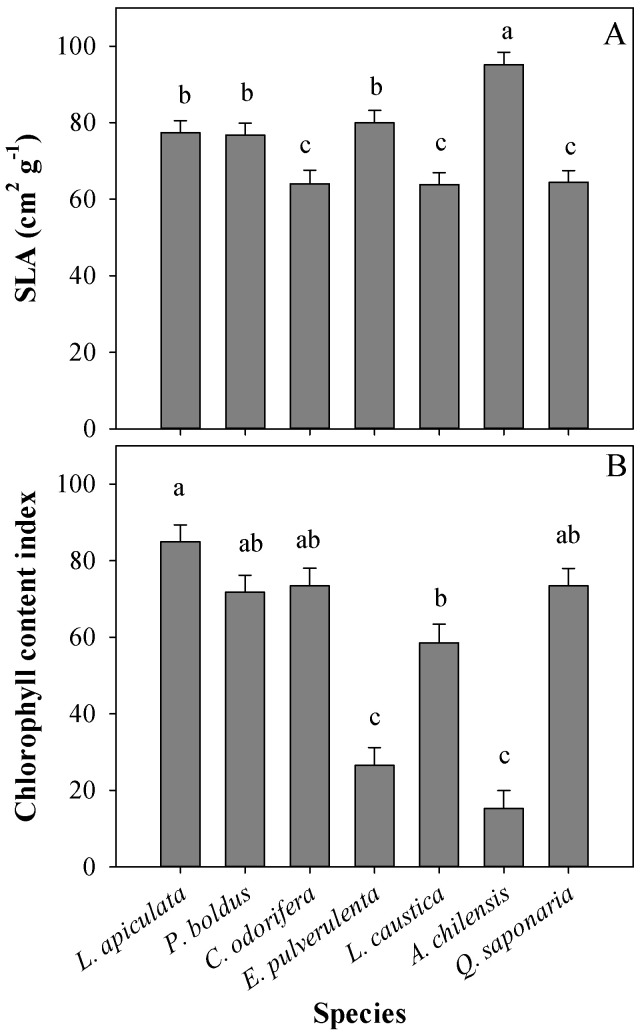
Mean specific leaf area (SLA) (**A**) and chlorophyll content index (**B**) per plant species. Different letters denote significant differences among plant species according to Tukey’s test, with a significance level of 0.05.

At the leaf level, significant differences in SLA were found among species (Table 1 and Figure 3A). *A. chilensis* had the highest SLA, whereas *C. odorifera*, *L. caustica*, and *Q. saponaria* had the lowest values, with *L. apiculata*, *P. boldus*, and *E. Pulverulenta* having intermediate SLA values (Figure 3A). There was also a main effect of the treatments on SLA (Table 1), with no differences between T0 (56 cm^2^ g^−1^) and T1 (61.6 cm^2^ g^−1^) and with a significant increase in SLA in treatment T2 (101.7 cm^2^ g^−1^). Similarly, species significantly differed in CCI, with *L. apiculata* having over 3-fold more chlorophyll than other species such as *E. pulverulenta* and *A. chilensis*, whereas other species had intermediate values (Table 1 and Figure 3B). We did not observe any effect of the treatments on CCI (Table 1, *p* = 0.0907). 

**Figure 4:**
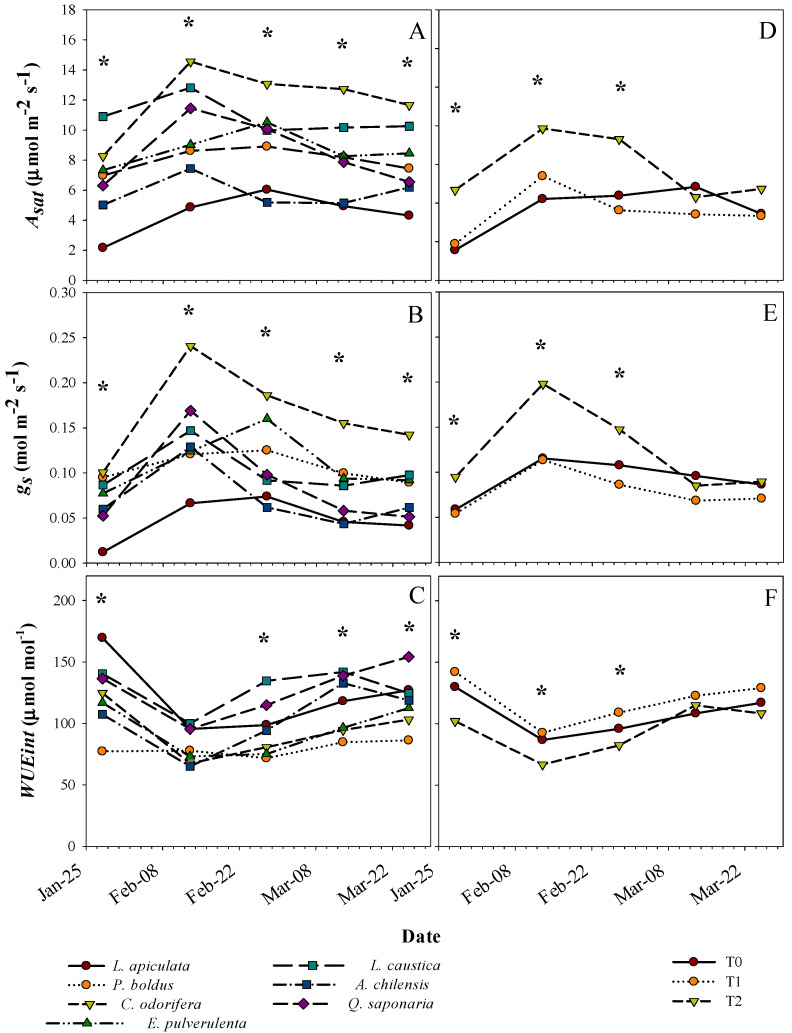
Means for net saturated photosynthetic rate (*A_sat_*), stomatal conductance (*g_s_*), and intrinsic water use efficiency (WUE_int_) over time per plant species (left panels) and treatment (T0, T1, and T2) (right panels). * indicates significant differences between species and treatments at a specific date, respectively, with a significance level of 0.05.

Species did not differ in the responses in gas-exchange parameters (*A_sat_*, *g_s_*, and WUE_int_) to the treatments (no treatment by species interaction) (Table 1). However, the differences found both among species and among treatments varied over time (Table 1 and Figure 4). *C. odorifera* and *L. caustica* tended to have the highest *A_sat_* over the period, which almost doubled the species with the lowest values, *L. apiculata* and *A. chilensis* (Figure 4A). For *g_s_*, *C. odorifera* and *L. apiculata* tended to have the highest and lowest values during the period, respectively (Figure 4B). Species such as *Q. saponaria* and *A. chilensis* had intermediate values of *g_s_* during February, but they had an important decline in this parameter towards the middle of March. Overall, peaks of *A_sat_* and *g_s_* occurred in early March for *L. apiculata, P. boldus*, and *E. pulverulenta* relative to those of the other species. 

**Figure 5:**
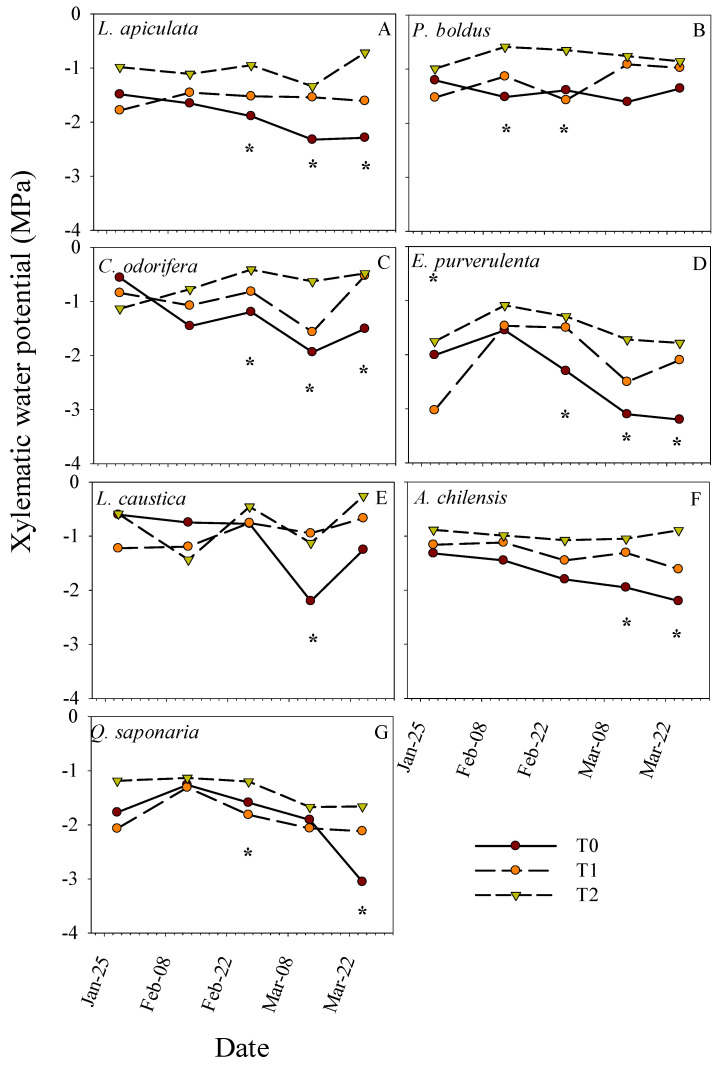
Means for midday xylematic hydric potential per treatment (T0, T1, and T2) over time and separated by species. * indicates significant differences between treatments at a specific date, respectively, with a significance level of 0.05.

On average, *P. boldus* had the lowest WUE_int_, where the highest values were observed in *L. caustica*, *Q. saponaria*, and *L. apiculata* (Figure 4C). *C. odorifera* and *E. pulverulenta* had almost the same WUE_int_ values across the period, being among the lowest along with *P. boldus*. Otherwise, general differences in gas-exchange parameters between treatments were expressed only on measurements during February (Figure 4D,E). Similar to other traits, treatments T0 and T1 had similar gas-exchange parameters, but they significantly differed from T2 at some dates (Figure 4D,E). *A_sat_* and *g_s_* were higher in T2 than in the other treatments (Figure 4D,E), whereas the opposite was observed for WUE_int_ (Figure 4F). *A_sat_* and *g_s_* were higher in T2 in February and decreased toward March, while in the other treatments, these parameters tended to be more stable across the period. Additionally, we found a differential response of the species in xylem water potential to the treatments across dates (Table 1 and Figure 5). Treatments differed in Ψ_x_ only on some dates, which vary with the species. The lowest and highest Ψ_x_ across the period were for treatments T0 and T2, respectively, whereas T1 tended to have intermediate values (Figure 5). The average Ψ_x_ for the last two dates (lowest values) for the control treatment T0 by species were, from low to high, E. *pulvurulenta* (−3.1 MPa), *Q. saponaria* (−2.5 MPa), *L. apiculata* (−2.3 MPa), *A. chilensis* (−2.1 MPa), *L. caustica* (−1.7 MPa), *C. odorifera* (−1.7 MPa), and *P. boldus* (−1.5) (Figure 5).

## 3. Discussion

The species assessed in this study are some of the most common in the sclerophyllous forests of the Mediterranean-type climate zone of central Chile, with some of them growing in close association depending on the geographic location [40]. Although this study was carried out at the nursery level, the experimental conditions mimic to some extent the establishment scenarios typically used in the restoration of this ecosystem type, which is the seedling establishment with some irrigation level and the incorporation of shading by using tree shelters. Overall, the evergreen species tested in this study exhibited great differences in growth and leaf morpho-physiological traits, but the way in which they responded to the treatments was similar, regardless of their shade or drought tolerance. Although a gradient was observed in the water stress imposed in the experiment, the acclimation response of the species was higher for shading than for the water restriction. 

Because the shading dome in T2 was built seven months before imposing the water restriction treatments, we assumed the plants were already acclimated to shade during this period. Then, during summer, the mesh ventilation in T2 cooled the air inside, especially at midday as has been reported by [28], creating a microclimate that decreased water and heat stress and favored better growth and physiological performance for both shade- tolerant and intolerant species. This is also supported by the lowest substrate temperature being recorded in T2 relative to T0 across the period (Figure 1), likely favoring root growth and functions. In *Vaccinuum* spp., Spiers [41] found that root and shoot growth was higher at substrate temperatures around 16 °C than at 27 °C and 38 °C, values that are in the range for the monitored treatments in our study. On the contrary, the black color of the pots exacerbated the highest substrate temperatures in the sun-exposed treatments (T0 and T1). Black pots significantly increase the substrate temperature, which consequently may decrease the root density in different species [42,43]. Therefore, although root growth was not measured, the lack of differences in the assessed traits among treatments T0 and T1 might be due to poor root development as a consequence of the high temperatures, which prevented seedlings from taking advantage of the extra water added in T1 relative to T0.

Although the period of water restriction in this study was short, it was applied during midsummer, when the higher air temperatures predominate. We are aware that pot studies do not represent the real conditions in the field, and in dry years, the water stress may start earlier in spring, extending the duration of the drought. However, because our interest was to characterize the short-term acclimation responses of the species to drought as a strategy to cope with the stress, we tried to exacerbate the intensity more than the duration of the stress. Several studies showed acclimation to drought in these types of sclerophyllous species in similar periods [20,21,22,24,44]. Therefore, the water restriction at T0 should have been enough to provoke the water stress on seedlings, especially since they were not previously acclimated to this driest condition. This is supported by the xylem water potential observed at the species level, where the most stressed seedlings were in T0 (i.e., lower Ψ_x_), followed by an ascendent gradient by T1 and T2. The fact that none of the species were able to efficiently use the extra amount of water in T1 relative to T0 may suggest that: (1) the degree of water restriction applied in T0 was just likely moderated and not enough to cause differences with T1; (2) the seedling stress by the irradiance and temperature in T1 (typically of potted studies as explained before) was high that was not alleviated by watering, which is supported by the great responses of the species to T2; or (3) seedlings used in the study were c.a., 70 cm in height with root collar diameter of c.a., 5 mm, which could have positively influenced the tolerance of T0 and T1 to water restrictions. Hence, all the species could hold their growth and physiological performance unaltered with water restrictions such as the one imposed by T0, with Ψ_x_ varying from −1.5 MPa in *P. boldus* to −3.1 MPa in *E. pulverulenta*. It is possible that the lower survival recorded in T0 relative to the other treatments was due to other ecophysiological processes not considered in this study, such as an increase in respiration rates by the highest temperature that led to a negative carbon budget, which needs further research. Although other studies in nursery and field trials have reported low pre-dawn potential for *C. odorifera* (−4.5 MPa, Montenegro and Riveros de la Puente [45]), *Q. saponaria* (−3.9 MPa, Donoso et al. [20]), *P. boldus* (−3.9 MPa, Peña-Rojas et al. [21]), *L. caustica* (−4.3 MPa, Peña-Rojas et al. [46]), and *A. chilensis* (−3.6 MPa, González-Villagra et al. [44]), it is not clear how species compare between them or how to use this information for management purposes, which was attempted in our study. A possible explanation for the low water potential reported by the latter authors could be the age and quality of seedlings at the beginning of their experiment in terms of height and diameter, which should be addressed in further studies. 

Height increments in the shaded treatment were 94% to 203% higher than in the other treatments, except in *P. boldus,* which showed no response (0%). This null response of *P. boldus* may be explained by the slow growth of the species and the need for a longer acclimation period, which likely goes beyond the experimental period considered in this study. Moreover, the high xylem water potential reached by *P. boldus* in T0 suggests that this species was less susceptible to water restriction in our study. Similarly, Peña-Rojas et al. [21] found no differences in height increments in the species in well-watered and water-restricted treatments (−0.56 MPa vs. −3.9 MPa, respectively). Moreover, Vogel et al. [47] found that growth and leaf production in the species were not altered by shading or irrigation after four seasons, which agrees with our findings. 

Evergreenness is a strategy that allows plants to grow the entire year [6], and in sclerophyllous species, it is an adaptation that also serves to cope with drought during summer [25]. Other adaptations of sclerophyllous species are reduced leaf area [48], stomatal control over transpiration [49], and the ease to extend roots towards new volumes of soil [50]. In our study, *L*. *caustica* and *Q*. *saponaria* are characterized by a deep root system as an important adaptation to survive the long summer periods [51,52,53]. However, both species exhibit no differences in height increment in the treatment T0, which corroborates that T0 imposed a moderate stress on the species under study. In addition, the fact that no species was found by treatment interaction for most leaf morpho-physiological traits (term ‘Trt × Spe’ in Table 2) suggests that the species under study have similar leaf acclimation to the stressor factors assessed, which is explained because all of them coexist under the same environmental conditions in Mediterranean-type climates. Among the most common traits reported as shade acclimation are an increase in SLA, an increase in chlorophyll content, and lower maximum net photosynthetic rates [27,33,34,54]. However, the magnitude of acclimation of different attributes and their phenotypic plasticity depend on the shade tolerance of the species [27], which did not coincide with our results since the leaf morphology of all the species was similarly affected by the treatments. In our study, the SLA in T2 almost doubled the values of the other treatments, whereas *A_sat_* was increased, and CCI showed no change with shading. The shading treatment intercepted approximately 80% of the photosynthetic active radiation (PAR), an interception value that is extremely high regarding the natural dispersion of sclerophyllous forests in central Chile and corroborates the need for sun protection at the early stages of development in the new environments created by the large-scale browning phenomenon on this ecosystem. This reinforces the phenotypic plasticity of all the species to shade at the seedling stage and that the lower *A_sat_* and *g_s_* in the sun-exposed treatments were likely limited due to other factors rather than water restriction, such as the excess of heat, irradiance, and substrate temperature in the pots. Moreover, we found a great variation in CCI among species (from c.a., 20 for species such as *E. pulverulenta* and *A. chilensis* to 85 for *L. apiculata*), which coincides with other studies showing high variation in chlorophyll content even in coexisting species [55]. Chen et al. [56] found a quick increase in chlorophyll content in response to shade (7 days) in tea leaves, whereas in our study, CCI was not increased by shading in any of the species. We argue that leaf chlorophyll content in evergreen species is not so sensitive to light compared to that in other species since leaves continue functioning over winter with low temperatures and light.

Overall, drought combined with excess light induces photo oxidation and decreases water potential, stomatal conductance, and chlorophyll content, with further consequences in CO_2_ assimilation [57,58]. On the other hand, Marchin et al. [59] mentioned that some broadleaf evergreen species increase their stomatal conductance under extreme heat conditions to prevent damage by leaf overheating, but this accelerates dehydration and turgor loss. Nevertheless, neither of the above-mentioned claims precisely represent the results found in our study. First, *A_sat_*, *g_s_*, and WUE_int_ did not vary between the water-restricted (T0) and well-watered (T1) treatments, likely for the above-mentioned explanations. Second, these parameters were relatively stable across the period in the sun-exposed treatments (T0 and T1), with higher fluctuations only in the shaded treatment, which reinforces that higher temperatures and excess of light are important factors limiting the physiological performance of these sclerophyllous evergreen species, even if water restriction is ameliorated. The values of gas-exchange parameters found in this study are in the range of other studies for some of the species, but especially for well-watered seedlings, with a considerable decrease in the parameters in more severe drought treatments [20,22,60]. Otherwise, it has been reported that sclerophyllous species with thicker and more-leathery leaves tend to have lower photosynthetic rates [6]. This was not the case for our findings, since, based on the SLA values, species such as *A. chilensis* would have thinner leaves (i.e., higher SLA) and lower *A_sat_*, whereas *C. odorifera* had the thicker leaves and the highest *A_sat_* among the species. 

The effect of shading and watering on seedlings’ growth in Mediterranean climates depends mostly on what practice may better alleviate water stress. Excessive light and temperature may exacerbate the water stress on seedlings and, consequently, survival [13], which is ameliorated by using shelters in restoration programs. Regarding the current climate scenario, shading seems to be critical at the establishment phase to promote stem growth for both shade-tolerant and -intolerant species in this type of semi-arid region, which agrees with other studies in nursery [54,61] and field conditions [36,62,63]. Studies on other Mediterranean zones show that the use of shading may be effective only for drought-tolerant species, even when seedlings receive some pulses of watering during summer [13,37]. Nonetheless, Quiroz et al. [62] found that the shade-intolerant temperate tree *Nothofagus alessandrii* Espinosa in central Chile had better growth and survival responses to shading (via using shelters) than to irrigation during the first 5 years after outplanting, supporting our findings that at early stages, there is a need for sun protection. Based on some limitations of our study, future research must consider a longer time of experimentation, more precise monitoring of water restriction, incorporation of more integrated measures of water stress such as carbon isotope discrimination and non-structural carbon analysis, and carbon partitioning, especially to assess the effect of temperature on the root formation of the different species.

Our results suggest that in environments with restricted water availability, shading positively influences the early growth and physiological acclimation of sclerophyllous species of the Chilean forests. From the restoration point of view, a specific management prescription at establishment could be the use of tree shelters. The positive effects exerted by tree shelters in the initial establishment of small seedlings in sites with water restriction and high radiances are largely known. In the case of watering, despite there being several options from deep pipes to dew harvesters [64], this technique is still expensive at an operational scale, especially if the proposed species are not of commercial interest. There are also uncertainties with the constant and sufficient water supply during the dry season. Instead of watering seedlings in the field, it would be more efficient to apply polymer gels [65] to support and secure the establishment of seedlings the first year after outplanting.

## 4. Materials and Methods

### 4.1. Plant Material and Study Design

The study was conducted at the nursery facilities at the Universidad de Talca, Chile (35° 24′ S, 71° 38′ W, 112 m. asl). In the fall of 2020, seedlings of seven tree sclerophyllous species (grown in 1 L plastic bags) were obtained from local commercial nurseries. The species corresponded to *Quillaja saponaria* Molina (Quillay), *Aristotelia chilensis* (Mol.) Stuntz (Maqui), *Peumus boldus* Molina (Boldo), *Lithraea caustica* (Mol.) Hook. and Arn (Litre), *Luma apiculata* (DC.) Burret (Arrayán), *Colliguaja odorifera* Molina (Colliguay), and *Escallonia pulverulenta* (Ruiz and Prav.) Pers (Corontillo). We checked that all seedlings were in good health conditions. Species life forms and functional groups (i.e., shade tolerance) are presented in Table 2. In these new nursery conditions, seedlings were acclimated for 1 month. Afterward, they were transplanted into 11 L black pots. At this stage, all seedlings were 3 years old and produced from seeds, except *A. chilensis*, whose plants were 2 years old and propagated vegetatively from 5 clonal genotypes. In the nursery, the experiment was established under outdoor conditions (i.e., environmental conditions of the site). The substrate corresponded to a mixture of decomposed pine bark (90%) and perlite (particle size 0.5–3 mm, 10%). We added 15 g per seedling of slow-released fertilizer (Basacote Plus 9M, Compo Expert S.A. Jalisco, Mexico), which provided macro- and micronutrients.

The experimental design was a split-plot design, arranged in a complete randomized design (CRD), with six replicates (single tree plot). Three treatments representing different scenarios at establishment in restoration programs were the whole plot, whereas the plant species were the split-plot (i.e., 3 treatments × 7 plant species × 6 replicates = 126 seedlings in total). Seedlings were separated enough to avoid potential shading between plants. The treatments were: T0: sun-exposed and water-restricted, T1: sun-exposed and fully irrigated, and T2: shaded and fully irrigated. The treatments were designed to assess the separated additive effect of watering and shading. All seedlings were equally watered to maintain the substrate at field capacity until the end of January 2021 (mid-summer), when the water-restricted treatment (T0) started. Afterward, T0 was watered at a dose of 250 mL of water every other day, whereas seedlings in the fully irrigated treatments (T1 and T2) were watered daily to substrate field capacity (700 mL per pot, approximately). This watering regime remained until mid-March and then was cut off. During the experiment, the substrate moisture and temperature were monitored on an hourly basis with a Thetaprobe soil moisture sensor (Delta-T Devices Ltd., Cambridge, UK) in the most extreme treatments, T0 and T2 (Figure 1). For the shading treatment T2, in winter 2020, seedlings were covered by a 14 m^3^ dome built with 80% black polyethylene mesh (Raschel^®^, Santiago, Chile) and secured with 1.3 cm diameter polyvinyl chloride (PVC). This gave six months of shade acclimation before the other treatments were imposed. We chose this type of net as it represents one of the cheaper options for building shelters in this ecosystem.

### 4.2. Growth and Physiological Measurements

Seedlings’ height was measured with a metric tape at the beginning and the end of the watering treatment application to obtain the increments in height (IncH). Since the application of the water restriction, leaf-level physiological measurements were obtained in five instances (from 28 January to 25 March 2021) on 4 plants per treatment and species combination. During this period, the average temperature and relative humidity were 18.5 °C and 71.8%, respectively. Net saturated photosynthesis rate (*A_sat_*, μmol m^−2^ s^−1^), stomatal conductance (*g_s_*, mmol m^−2^ s^−1^), and intrinsic water use efficiency (WUE_int_, μmol mmol^−1^) were measured for one fully expanded leaf in the upper third of the plant, using a portable gas-exchange system LI 6800 (LICOR Inc., Lincoln, NE, USA). Initial chamber conditions were set up at ambient conditions during measurements, with a temperature of 20 °C, CO_2_ concentration of 400 ppm, relative humidity of 50%, and PAR of 1800 µmol m^−2^ s^−1^. Measurements were performed between 09.00 and 12.00 h at local time. On the same dates, midday xylem water potential (Ψ_x_) was measured on one leaf using a Scholander pressure chamber (PMS Instrument Co., Albany, OR, USA). Before the measurements, the attached leaves were covered with a plastic sheet and wrapped in aluminum paper to equalize the water potential between the leaf and stem. Additionally, at each date, we measured the chlorophyll content index (CCI) using the chlorophyll meter MC-100 (Apogee Instruments, Inc., Logan, UT, USA). At the end of the growing period, we collected foliage from the different seedlings, which were scanned to determine leaf area using the software Image J 1.46r (developed by the National Institute of Health). Then, the foliage was oven-dried at 65 °C to a constant weight to determine the specific leaf area (SLA, leaf area to dry weight ratio).

### 4.3. Statistical Analysis

Data analyses were made with the PROC MIXED procedure of the software SAS 9.4 (SAS Institute Inc., Cary, NC, USA). We used a split-plot design analysis for more integrated measurements such as IncH and SLA, with factor treatment, species, and their interaction being considered fixed effects. For measurements monitored through time (leaf physiological traits and CCI), we expanded the model to a repeated measures analysis, with the factors treatment, species, date, and the interaction considered fixed effects. To improve the inference over the repeated measures analysis, preliminarily for each trait we modeled different variance–covariance structures (compound symmetry, unstructured, and first-order autoregressive) for the residual effects and selected the best model according to the Akaike information criteria. When needed, we used Tukey’s test for mean multiple comparisons. Significant differences were considered at a probability level of 0.05.

## 5. Conclusions

The evergreen sclerophyllous species tested in this study exhibited great differences in growth and leaf-morpho-physiological traits. However, the way in which they responded to the treatments was similar in six of the seven species, regardless of their shade or drought tolerance. *P. boldus* was the exception, and its null response in growth to the treatment was likely due to its slow growth and the need for a longer period of acclimation to the stressors factors. Moreover, species acclimation was higher for the shading than for the watering treatments. Given the excess of temperature and light in the Mediterranean zone of central Chile, the use of shading (i.e., via shelters) should be mandatory for these species; otherwise, irrigation might be insufficient to secure seedling establishment. Regarding the high costs involved in ecosystem restoration, species-specific responses to establishment treatments must be identified to increase the success of seedling establishment after outplanting, first with screening trials in nursery experiments and then validation in field conditions.

## Figures and Tables

**Table 1:** *p*-values on the analysis of variance for increment in height (IncH), specific leaf area (SLA), chlorophyll content index (CCI), light-saturated photosynthetic rate (Asat), stomatal conductance (gs), intrinsic water use efficiency (WUEint), and xylematic hydric potential (Ψ_pot_). Significant effects (*p*-values < 0.05) are shown in bold font.

Source of Variation	IncH	SLA	CCI	Leaf Physiological Traits			
				*A_sat_*	*g_s_*	WUE_int_	Ψ_pot_
Trt	**0.0026**	**<0.0001**	0.1149	**0.0123**	**0.0166**	0.0716	0.0867
Spe	**<0.0001**	**<0.0001**	**<0.0001**	**<0.0001**	**<0.0001**	**0.0003**	**<0.0001**
Trt × Spe	**0.0435**	0.3035	0.0907	0.1279	0.4162	0.6283	**0.0004**
Date			0.3295	**<0.0001**	**<0.0001**	**<0.0001**	**<0.0001**
Trt × Date			0.4978	**0.0005**	**0.0003**	**0.0103**	**<0.0001**
Spe × Date			0.0831	**0.0273**	**0.0003**	**0.0054**	**<0.0001**
Trt × Spe × Date			0.5464	0.1015	0.5234	0.3456	**0.0071**

Note: Trt: experimental treatment, Spe: plant species.

**Table 2:** Life-form and functional group of the species under study.

Species	Family	Life-Form	Functional Group
*Luma apiculata* (D.C.) Burret	Myrtaceae	Evergreen Tree	Shade-tolerant Drought-sensitive
*Peumus boldus* (Molina)	Monimiaceae	Evergreen Tree	Drought-tolerant Semi-shade-tolerant to intolerant
*Colliguaja odorifera* (Molina)	Euphorbiaceae	Semi-deciduous Shrub	Shade-tolerant Drought-tolerant
*Escallonia pulverulenta* (Ruiz and Pav.) Pers.	Escalloniaceae	Evergreen Shrub	Shade-intolerant
*Lithraea caustica* (Molina) Hook and Arn.	Anacardiaceae	Evergreen Tree	Shade-tolerant Drought-tolerant
*Aristotelia chilensis* (Molina) Stuntz	Elaeocarpaceae	Evergreen Tree	Shade-intolerant Drought tolerant
*Quillaja saponaria* (Molina)	Quillajaceae	Evergreen Tree	Shade-intolerant Drought-tolerant

## Data Availability

Data are contained within the article.
